# Medical Education

**Published:** 1868-06

**Authors:** J. S. Jewell

**Affiliations:** Chicago, Pres’t of the Association


ARTICLE XIX.

### MEDICAL EDUCATION.—AN ADDRESS DELIVERED BEFORE THE ALUMNI ASSOCIATION OF CHICAGO MEDICAL COLLEGE.

BY J. S. JEWELL, M.D., of Chicago, Pres't of the Association.

Delivered at the Annual Meeting of the Association, held at Chicago Medical College, March 3d, 1368.

GENTLEMEN OF THE ALUMNI ASSOCIATION OF CHICAGO MEDICAL COLLEGE:

The institution of which we are members, has just closed the ninth year of its existence. Beginning under external circumstances not the most auspicious, it has passed evenly and steadily along, to the surprise, even of many of its friends, not to mention those who looked upon it, if not with enmity, yet as a spasmodic or Utopian scheme. In spite of adverse circumstances, unfriendly and often unbecoming criticisms, it has, from the first, enjoyed a real, healthy growth, and to-day has an acknowledged and a permanent place among the institutions of our city and country; one, *we* may be permitted to say, in which is found only cause for congratulation. From a beginning without means, it has advanced to a position where its foundation has been, we trust, enduringly laid. To whatever other causes this success has been due (and there *are* other causes), it is in great measure due to the men who first founded it, to give practical form to their well-grounded views in relation to medical education, and to which practical effort they have consecrated no small portion of their time, means, and influence, without any corresponding material equivalent as a return. To no one of that number can these remarks be so fully applied as to the worthy and respected *President* of our *Alma Mater.*

By many of us was it thought to be a fitting time to call the children, who have been scattered abroad, back to the old family ground, for mutual advice, encouragement, and congratulation. With nine years of our institution committed to history; with a band of faithful and generous alumni; and with the prospect and determination, if practicable, of taking new and important steps in relation to medical education; and in view of the necessity of harmony of purpose and effort, so far as the alumni are concerned, you have been called together. We had hoped to have seen a greater number present from the classes of former years; but to such as are with us, and to the new members, in behalf of the faculty, trustees, and resident alumni, I extend you a welcome, which lacks language corresponding to its cordiality.

This coming together is not for purposes of self-glorification, though we may in all propriety congratulate each other and the institution, on the success it has achieved. It is rather for the purpose, as I view it, of renewing old and forming new associations; rekindling our zeal; perfecting our organization, in view of its ends; and of considering, at this juncture, what are the present relations of the institution, in the welfare of which we have so deep an interest—to the profession and medical education—and what should or will be its policy in the future, and in what way, if any, we, as its members, may aid its efforts in the worthy cause to which it is committed. Never, in its history, could a meeting of its graduates and friends, with such purposes, take place with more propriety.

Then, in the *first* place, it is only necessary to remind you, I hope, how much an institution, especially one like this, is dependent on its graduates. They are its living, acting, practical testimonies; epistles known and read of all men. If we are progressive, liberal, and practical in spirit, comparatively accurate and extensive in professional learning, and faithful in the discharge of professional and other duties, the result cannot but be to reflect honor back on the institution from which we have issued. Conversely, if we display opposite qualities, the institution, by an inevitable consequence, shares our failure or disgrace. Thus, in some important respects, our interests are *one*—the relations reciprocal. You know not with what pleasure and satisfaction the institution, represented in its faculty, looks out after those it has fostered and upon whom it has set the seal of its approbation. How they watch with a just pride their good deeds, and with what pain they learn of any act inconsistent with a true manhood. They cannot but feel anxious, since the responsibility is recognized and accepted, which rests on them, in sending out members to assume responsible places in the communities of our land.

The impressions of various kinds made on the mind of the student, while passing through the prescribed course, cannot be devoid of influence on his subsequent professional career. He will have, as a general thing, a broad, manly, and liberal, or a narrow, low, and sordid view of his profession; much in accordance with the spell cast about him by the institution to the guidance of which he has committed himself. These interdependent relations should never be forgotten, and I hope they are remembered by all congregated here to-day.

In the *second* place, let us not forget the relations our institution sustains to medical education and the profession in this country, *to-day*, not less than through its entire history. That we may understand the relation just mentioned, let us glance briefly at its two terms. But *first*, the *state of medical education* in general, and the *needs* of the profession.

Some of you will be surprised to learn, that just one hundred years ago, almost to a day, the first medical school was established in this country. This was in 1768, in the city of Philadelphia, and *now we* meet *here* on the threshold of a new century! That school and others, called into existence sooner or later, were evidently intended to meet, temporarily, an emergency felt in a new country. It does not seem to have entered the thought of these pioneers in medical education that their primitive schools were adequate to the *real* wants of the profession. Men *still* returned to the *old* world, to acquire what the imperfect schools of the *new* could not afford. But so pressing were the needs of the new communities, and so great the corresponding inducements to enter practice, that, in connexion with the difficulty and expense of visiting the old world, it soon came to pass that the profession, as a whole, had only such attainments as the imperfect home schools could afford. The practical character of a people devoted, necessarily, at that early period, to the development of the material resources of the country, was soon communicated to the profession, with the effect to render men careful only of securing a mere practical acquaintance with the simple diseases prevalent in the early settlements. Then, the absence of the legal restrictions which, from time immemorial, were in force in the old world, by which communities were in some measure protected from quacks and pretenders, permitted all persons whom inclination, a sordid interest, or accident led to practice, to do so without let or hindrance. This opening the professional arena to a host of incompetent and quackish persons; this practical obliteration of all well-marked distinctions in the popular mind, or even in the profession, between incompetent professionals and the educated physician was striven against, but with indifferent success. Those who took this stand, gradually abandoned the unequal contest and soon came to tolerate, if not to fall in with, the adverse tide. The final result was, a majority of the practicing physicians in the country had never beheld the inside of a medical college, even such as the country afforded, and among the remainder, the majority had attended but one course of lectures before becoming involved in the *toils* of practice; while the few only, as rare exceptions, had a diploma from an indigenous college. The rarest professional phenomenon was to see, or even *hear* of a man who had graduated at one of the renowned schools of the old world. This state of things, true to the letter, led to several results. Among them, we notice:

1. A miserably low standard of attainment among those popularly accounted as members of the profession. The result, in fact, was to abolish *any* valid standard for comparison and judgment.

2. A continuance, as a permanent system, with occasional unimportant changes for better or worse, of the temporary scheme adopted in the beginning, in relation to *course*, *time*, and *range* of study in the medical colleges.

3. A most overwhelming admiration for the *men*, *institutions*, and *books* of the old world. This latter is, even now, only *beginning* to reäct to something like just proportions. These, in brief, were the main results reached almost a century ago.

The one hundred years which have elapsed since the establishment of the first medical college, have witnessed achievements in this fair land of a development and progress in the material, scientific, educational, moral, and religious aspects of life, such as must be, and is, matter for unfeigned astonishment to every reflecting mind. Everywhere, forgetting the things that are behind, has marked progress been made toward practical perfection. But one exception, however, must be made to this statement, and *only* one. It relates to our profession. We do not make this as a general statement. We do not refer to the progress of medical knowledge, because that has kept pace at least with other progressive forms of knowledge. Neither do we refer to the state of the *professional mind* as to progress in medical education, because there has been decided advance in this respect. While all forms of science, and especially those tributary to medicine, have been widening their fields and multiplying their objects an hundred fold, medical schools have, almost without an exception, stood *absolutely still.* Generally speaking, they have revolved within the same limits and on much the same ground they did *one hundred* years ago. There has been no advance, in any sense, corresponding to what has been observed in other departments of education, or commensurate with the growing state of medicine. This has not existed because the profession was unaware of it, or had not protested against it. Protestations were often made, and the more so as time passed on. There has been a progressively increasing demand on the colleges for higher education; but the demand, practically speaking, has been unheeded. But what *has* been actually the practice of the schools in this matter, and what *is* the practice *now?* It is so well known as almost to weary one to hear it stated. The colleges have almost uniformly laid down two courses of four calendar months each, or sixteen weeks; in all, something like three years is supposed to be spent in study before taking a degree. It is really amazing to look over the details of the curriculum and see how much must be crowded in the brief space of three years. But in some cases, there has been ever a letting down from the positions assumed just one hundred years ago. The candidate was *then* obliged, as he is not now, to submit to examinations in *latin* and *mathematics*, and present and defend a *latin thesis*, besides pursuing and submitting to examinations in all medical subjects, properly so-called.

Though there has been such a rapid extension of the domain of medicine, there has been no corresponding extension of college terms. As they were one hundred years ago, so are they, generally speaking, now. This remarkable brevity in number and length of college terms is not compensated for by any extended and accurate preliminary culture, as a rule, in attaining which, valuable habits of study are formed and acquirements made. As often as any other way, students come from the farm, or some other sphere in which they seldom possess any of the above prerequisites. But, perhaps, these defects in the system are counterbalanced by the adoption of a happy logical order among the various studies, so as to economize the time? It would surprise any *disinterested* but intelligent observer to be told, as he must be in truth, that faulty as the system is in other respects, it is most so here. There has been, as a *rule*, no kind of attention paid to a logical order among the various branches taught. *Order* is said to be the *first* law of heaven. I do not know about that; but this I *do* know, it is likely to prove the *last* in medical college instruction. This cannot be set aside, as of small consequence. Everywhere else, in all kinds of business, in learning all other professions, in all other forms of teaching or education a *progressive order* is considered, and justly so, as an indispensable, almost a *paramount* feature. But in medicine, instead of *this*, its various branches have generally been presented in the veriest medley. We cannot plead that medical is an exception to other kinds of teaching; neither can we plead custom, or authority, or respectability, such subterfuges will not do. It will be no offset to this to say, our institutions have turned out men who have become eminent. This is but the transparent logical fallacy of "*post hoc, ergo propter hoc.*"

It is a mortifying, indisputable fact that, in this respect, the medical colleges in this country are behind the times. There is but one course open which should be entertained. That is, enlightened, liberal *reform.* This should be pushed, in spite of the harmless sneers of a halting conservatism. The time *has* been, when it was best to *wait* and consider, *perhaps*, but that time has passed. There is no longer any valid excuse for delay. But what effect on the profession and on the progress of medicine has this unfortunate attitude of the colleges exerted? As to the former, certainly not good. No one will contend but if the colleges had exacted three terms instead of two, of six months each instead of four, medical men, as a whole, would have been better educated. No one will contend that the colleges have no responsibility in this matter. As water will not rise above its fountain head, neither will the profession, as a *body*, transcend its fountains of instruction and intelligence. This is true, if there is any force in personal influence and example in the affairs of men. As to the latter—the progress of medicine—the same may be said. It savors of a *truism*, to say that medicine would have been advanced more than it has been, if the profession had been more learned, capable, and enthusiastic, as it would have been under a different.course of instruction. These effects on the profession and the progress of medicine, have not been passing unperceived. Whenever the profession has been brought to reflect, as it often has, and when the opportunity has been offered for expression, almost invariably the call has been for higher education. The reports of societies and medical journals have often been fairly burdened with unmistakable expressions, that the colleges must adopt a more elevated and rational system of instruction. It is almost painful to think in what estimation our system is held abroad. To our complacent, minutely furnished, and highly favored brethren in Europe, we are little better than gentiles or barbarians, in a *scientific* point of view. Every one knows how lightly the title of doctor is held in popular esteem in this country, and how narrow, for reasons at once obvious, is the line between quacks and the regular profession. Is it any wonder to the *frank* and intelligent outsider, that mistakes should be made by the people as to where that line is, if it exists? Having glanced briefly at the relations of medical colleges in *general* in this country to medical education, let us notice, in a few words, what are and have been, from its beginning, the relations of this institution to the same.

The establishment of this school took place under the conviction, in the minds of its founders, that there was no longer any excuse for not making *some* change in the right direction. While the true conception required to satisfy it a sweeping and radical reform, yet the practical difficulties in the way of the enterprise made it necessary to *lower* the conception for the time, without, however, losing sight of it. The three features of importance the design included, were:

1. To lengthen the term to five instead of four months.

2. Multiply the chairs, by dividing some of the leading ones, as anatomy, chemistry, surgery, practical medicine, etc., that each teacher might have a narrower field, and more time thoroughly to occupy it; and the *student* more time and less confusion for *study*.

3. To arrange the various branches embraced in the curriculum, so that the most primary, as determined by the real wants of the student, should come first, while others should follow in a strictly natural or logical order; so that the studies of *this* day, week, or session would not be alone for themselves, but *preparatory* to ulterior studies, and so on to the end of the curriculum. The student was to be restrained from entering on that part of his course to-day, which, to be comprehended, would remand him to-morrow to some prior study he had neglected, or, worse still, which it had been *arranged* to lead him into at some subsequent period.

You are all familiar with the division of studies and course, in conformity with the above, which has, from the beginning, prevailed in this school. While it was felt that the *final* step could not be taken; that the contemplated change must be gradual; yet that the extension of time from four to five months, and the division of the chairs adopted, and the attempt to systematize the course were important advances in a normal direction, none will deny. The ground at first taken has not since been abandoned. So far from any disposition to this being manifested, the faculty, or various members of it, have informally had under consideration various schemes for attaining higher ground in relation to medical education. Time passed on, and it began to be known that a medical college planted on these principles could be sustained, and was actually receiving sympathy and encouragement from the profession, so far as it became known.

At each meeting of the American Medical Association, medical education received thoughtful attention, until at last, at the meeting of 1866, held in Baltimore, it culminated in the appointment of a committee, which was empowered to call a convention of colleges, to consider the whole matter of medical college instruction, with a view to reform. In obedience to the call of the committee, a convention assembled at Cincinnati, beginning on the 3d of May, 1867, Prof. Alfred Stillé, of Philadelphia, presiding. That convention continued its sessions for three days, and was made up of delegates from most of the prominent colleges in the United States. *This* institution was worthily represented by Professors Davis and Byford. On the first day of the convention, the former presented propositions for its action, and, during its continuance, ably supported them. The meeting seems to have been conspicuous for its harmony and good feeling, and the propositions were thoroughly, earnestly, and temperately discussed. The final result, was the adoption of the propositions at first submitted, without any fundamental change, and without a dissenting voice. They read, omitting the last of the series, as follows:

"*Resolved:*

"1st. That every student applying for matriculation in a Medical College, shall be required to show, either by satisfactory certificate, or by a direct examination by a Committee of the Faculty, that he possesses a thorough knowledge of the common English branches of education, including the first series of mathematics, the elements of natural sciences, and a sufficient knowledge of Latin and Greek to understand the technical terms of the profession; and that the certificate presented, or the result of the examinations thus required, be regularly filed as a part of the records of each Medical College.

"2d. That every Medical Student shall be required to study four full years, including three regular annual courses of Medical College instruction, before being admitted to an examination for the degree of Doctor of Medicine.

"3d. That the minimum duration of a regular annual lecture term, or course of Medical College instruction, shall be six calendar months.

"4th. That every Medical College shall embrace in its curriculum the following branches, to be taught by not less than nine Professors, namely: Descriptive anatomy, including dissections; physiology and histology; inorganic chemistry, materia medica, organic chemistry, and toxicology; general pathology, therapeutics, pathological anatomy, and public hygiene; surgical anatomy and operations of surgery; medical jurisprudence and medical ethics; practice of medicine, practice of surgery, obstetrics, and diseases of women and children; clinical medicine and clinical surgery. And that these several branches shall be divided into three groups or series, corresponding with the three courses of Medical College instruction required.

"The first, or *Freshman Series*, shall embrace descriptive anatomy and practical dissections; physiology and histology; inorganic chemistry and materia medica. To these, the attention of the student shall be mainly restricted during his first course of Medical College instruction, and in these he shall submit to a thorough examination, by the proper members of the Faculty, at its close, and receive a certificate indicating the degree of his progress.

"The second, or *Junior Series*, shall embrace organic chemistry and toxicology; general pathology, pathological anatomy, therapeutics, and public hygiene; surgical anatomy and operations of surgery; medical jurisprudence and medical ethics. To these the attention of the medical student shall be directed during his second course of Medical College instruction, and in them he shall be examined, at the close of his second course, in the same manner as after the first.

"The third, or *Senior Series*, shall embrace practical medicine; practical surgery; obstetrics, and diseases peculiar to women and children; with clinical medicine and clinical surgery in a hospital. These shall occupy the attention of the student during his third course of College instruction, and at its close he shall be eligible to a general examination for the degree of Doctor of Medicine.

"The instruction in the three series is to be given simultaneously, and to continue throughout the whole of each annual College term; each student attending the lectures on such branches as belong to his period of progress in study, in the same manner as the sophomore, junior, and senior classes, each pursue their studies simultaneously throughout the collegiate year, in all our Literary Colleges.

"5th. That every Medical College should immediately adopt some effectual method of ascertaining the actual attendance of students, upon its lectures and other exercises, and at the close of each session, of the attendance of the student, a certificate, specifying the time and the courses of instruction actually attended, should be given, and such certificate only should be received by other Colleges as evidence of such attendance."

Immediately following the convention, the American Medical Association convened on the 7th, at the same place, and on the second day Dr. Sayre, of New York, offered the following:

"*Resolved*, That this Association, most heartily approving the whole action of the Convention of Medical Colleges, *urge its practical adoption on* ALL the Medical Colleges in the country."

This resolution was adopted. It will be observed by you all, that the course, finally agreed on in the Convention and approved by the Association, differs only in *measure*, not in *kind*, from that adopted in this College from the beginning, and which has been urged for years past, on all occasions, by the worthy President of this faculty, to whose action, more than that of any single man, the movement now under consideration may be fairly attributed.

It is on the eve of this grand advance determined on among the colleges, and upon which the profession has set the seal of its approbation, and which is to open the new century dawning on American medical colleges; it is to catch the spirit and feel the spell of this medical college centennary; to approve and urge, in our proper capacity, the adoption of the propositions agreed on; to exchange congratulations; renew old associations and form new; rekindle our zeal, and consecrate ourselves anew and intelligently to the best interests of the profession; it is for *such* purposes we have come together to-day. To these calls may we all respond. Let us not leave here, faltering as to our personal duty, however humble, nor devoid of hope or interest in this cause. Let us try and be worthy of, and display a truly filial affection for, our Alma Mater, acquitting ourselves in whatever sphere as men, and ever ready to come up and lend what assistance and encouragement we may in forwarding the noble cause our Institution is so honorably identified with.

